# Prospects and limitations of improving skeletal growth in a mouse model of spondyloepiphyseal dysplasia caused by R992C (p.R1192C) substitution in collagen II

**DOI:** 10.1371/journal.pone.0172068

**Published:** 2017-02-09

**Authors:** Machiko Arita, Jolanta Fertala, Cheryl Hou, James Kostas, Andrzej Steplewski, Andrzej Fertala

**Affiliations:** Department of Orthopaedic Surgery, Sidney Kimmel Medical College, Thomas Jefferson University, Philadelphia, Pennsylvania, United States of America; Universite de Lyon, FRANCE

## Abstract

Skeletal dysplasias form a group of skeletal disorders caused by mutations in macromolecules of cartilage and bone. The severity of skeletal dysplasias ranges from precocious arthropathy to perinatal lethality. Although the pathomechanisms of these disorders are generally well defined, the feasibility of repairing established aberrant skeletal tissues that developed in the presence of mutant molecules is currently unknown. Here, we employed a validated mouse model of spondyloepiphyseal dysplasia (SED) that enables temporal control of the production of the R992C (p.R1192C) collagen II mutant that causes this disease. Although in our earlier studies we determined that blocking the expression of this mutant at the early prenatal stages prevents a SED phenotype, the utility of blocking the R992C collagen II at the postnatal stages is not known. Here, by switching off the expression of R992C collagen II at various postnatal stages of skeletal development, we determined that significant improvements of cartilage and bone morphology were achieved only when blocking the production of the mutant molecules was initiated in newborn mice. Our study indicates that future therapies of skeletal dysplasias may require defining a specific time window when interventions should be applied to be successful.

## Introduction

During prenatal development, various collagen types contribute to shaping the embryonic cartilaginous skeleton and maintaining its structural integrity. This defines the spatial organization of chondrocytes whose biological activities control bone growth. In a postnatal growth plate, chondrocytes arrange into columns within which specific cell subpopulations form the resting, proliferating, pre-hypertrophic, and hypertrophic zones. These chondrocytes interact, via receptors, with the extracellular matrix (ECM) of the growth plate to maintain this arrangement, thereby supporting the proper growth of bones that develop by endochondral ossification [1, 2].

Among the cartilaginous structural macromolecules of developing bones, collagen II is the most abundant. Documented aberrations of skeletal growth due to mutations in *COL2A1* confirm the key role of collagen II in bone development [3]. Collagen II mutations produce diverse chondrodysplasia phenotypes classified as spondyloepiphyseal dysplasia (SED; OMIM 183900). SED variants range in severity from achondrogenesis type II, which is lethal at or before birth, to late-onset SED with precocious osteoarthritis [3]. Molecular-level consequences of different mutations in collagen II may produce misfolded mutant collagen II molecules with lower thermostability of the collagen triple helix, and cause abnormal fibril formation. Studies demonstrated, that abnormal collagen II fibrils are not able to interact properly with other elements of the ECM [4]. Thus, mutations in collagen II have a broad negative impact on the architecture of the cartilage’s ECM. Moreover, mutations in collagen II may increase intracellular accumulation of mutant chains and molecules that interact with them, *e*.*g*. fibronectin, hence causing endoplasmic reticulum (ER) stress [3, 5].

To date, no therapies exist that effectively target the molecular basis of heritable skeletal dysplasias. Experimental approaches to reduce the consequences of collagen mutations associated with skeletal dysplasias include cell therapies, gene therapies, and therapies to reduce ER stress. Researchers have also tested the utility of growth hormone and statins to improve the growth of bones in skeletal dysplasias [6, 7].

Initial clinical studies on the therapeutic utility of cell transplants from healthy donors targeted patients with severe forms of osteogenesis imperfecta (OI), a bone displasia mainly caused by mutations in collagen I [8–10]. Both postnatal cell infusions and prenatal infusions were tested in the OI patients [11–13]. These studies found that the infusions had only transient positive effects on linear growth. Since the studies only detected a fraction of a percent of the donors’ cells in the bones of the recipients, the true potential of the cell-based strategies is difficult to assess [10].

Reseachers have also explored gene therapies for treating OI. Gene therapies, which focus on allele-specific gene silencing, employ antisense oligonucleotides, hammerhead ribozymes, and siRNA, among other agents for targeting mutant transcripts [14]. While blocking these transcripts was somewhat effective in cell culture systems with collagen I mutations, the clinical applications of gene therapies for treating SED remain challenging [15–17].

In another approach, scientists have suggested that reducing ER stress, which contributes to the pathology of skeletal dysplasias, may improve the development of bones harboring mutant proteins [18–21]. Scientists have attempted to reduce ER stress by utilizing chemical chaperones that promote the folding of misfolded collagen mutants, thereby improving their thermostability and secretion from cells. Studies have demonstrated treatment benefits with chemical chaperones 4-phenylbutyrate (PBA), glycerol, and trimethylamine N-oxide (TMAO) [22–24].

Other efforts to treat chondrodysplasias have included clinical test with the use of growth hormone. These efforts, however, did not show any significant improvements in patients with achondroplasia (ACH), hypochondroplasia, SED, and pseudoachondroplasia (PSACH) [6]. In contrast, applying statins in ACH model mice led to a significant recovery of bone growth [7].

Despite these therapeutic approaches, however, the ability to prevent development of dysplasia phenotype and to restore proper growth with therapeutic interventions remains elusive. Similarly, the stage of skeletal development at which therapeutic interventions should be applied in order to be effective has not yet been clearly defined. Consequently, to study these problems, we employed an inducible mouse model of mild SED caused by the R992C substitution in collagen II [25]. Because the mutant transgene may be switched off at any developmental stage in this model, we were able to study the consequences of stopping the expression of the mutant collagen II chains. Our earlier studies thoroughly validated this model and demonstrated that maintaining the expression of the R992C collagen II constantly alters the structure of the growth plates that harbor this mutant. We also showed that switching off the expression of the mutant at the beginning of embryonic growth blocks the SED phenotype [25].

In this study, we analyzed the consequences of switching off the R992C collagen II expression at different postnatal stages. Since switching off the expression of the R992C collagen II chains completely eliminates their influence on growing bones, our model represents an ultimate therapeutic approach. Based on our findings, effective strategies to prevent or repair skeletal aberrations caused by mutations in collagen genes may depend on the specific time when they are applied.

## Materials and methods

### Mutation nomenclature

The R992C (p.R1192C) amino acid substitution is named according to the literature, with amino acid residues numbered from the first glycine residue of the collagen triple helix.

### Transgenic mice

All mice received humane care according to the guidelines in the Guide for the Care and Use of Laboratory Animals. Procedures performed on animals were approved by the Thomas Jefferson University’s Institutional Animal Care and Use Committee.

We employed a validated mouse model of SED in which a construct encoding procollagen II with the R992C substitution is expressed conditionally; endogenous *Col2a1* gene is expressed constantly [25]. To facilitate direct microscopic assays of the expression patterns of the transgene, the chains of the exogenous procollagen II were tagged with green fluorescent protein (GFP), a tag that is naturally cleaved off together with the C propeptides [5, 25]. GFP-tagged procollagen II molecules are referred to as ProGFP.

Key characteristics that define the model mice are: (i) in addition to DNA construct for the R992C-ProGFP, the tetracycline (Tet) transactivator (tTA) expression is maintained in the mice to achieve Tet-dependent regulation of expression; (ii) the presence of cre recombinase, the expression of which is driven by a chondrocyte-specific promoter (Col2a1-cre), facilitates the chondrocyte-specific expression of collagen II transgenes; (iii) the presence of all three transgenes (*i*.*e*., for the R992C-ProGFP; for tTA; and for Col2a1-cre) is needed for expression of the exogenous R992C-ProGFP construct; (iv) the expression of the R992C-ProGFP is active in the absence of doxycycline (Dox) while the expression is inhibited completely in the presence of Dox supplied in drinking water at 0.2 mg/ml; (v) triple-transgenic mice expressing the R992C-ProGFP, together with tTA and Col2a1-cre, are described as R992C-ProGFP(+); (vi) because of the genetics, not all littermates are positive for all three transgenes needed to produce the R992C mutant, transgenic mice that lack at least one of the required three transgene DNA constructs (*i*.*e*., either for the R992C-ProGFP, for tTA, or for Col2a1-cre) are named R992C-ProGFP(-); in these mice, the exogenous R992C-ProGFP protein is not produced; and (vii) the R992C-ProGFP(-) mice are considered phenotypically wild type [25].

### Analysis of triple transgenes

Offspring generated via a breeding strategy described elsewhere were analyzed by PCR for the presence of the tTA, the Col2a1-cre, and the R992C-ProGFP constructs. Note that the theoretically predicted percentage of the triple-transgene offspring generated with the breeding protocol employed here is 12.5% [25].

### Regulation of transgene expression and experimental groups

Initially, in triple-transgene R992C-ProGFP(+) embryos, the R992C mutant was produced during development because pregnant mothers did not receive Dox. In the R992C-ProGFP(-) members of the same developing litter that did not harbor all three transgenes, however, this mutant was not produced. After birth, Dox treatment was initiated in newborn mice (a NB-Dox group), in one-week-old mice (a 1w-Dox group), or in two-week-old mice (a 2w-Dox group) to block the expression of the R992C-ProGFP. Starting from those time points, Dox was supplied constantly until the mice were sacrificed at 7-week-old or 10-week-old time points. While the 7-week time point was chosen to analyze selected features of the growth plates in developing bones, the 10-week time point was chosen to analyze corresponding features in mature bones [26–29].

In addition to the NB-Dox, 1w-Dox, and 2w-Dox groups, in some experiments, we also included a control group of mice developed in the absence of Dox. In this group, littermates harboring three transgenes produced the R992C mutant constantly, while the littermates missing at least one transgene did not produce this mutant at all.

R992C-ProGFP(+) and R992C-ProGFP(-) mice developed in the presence of Dox are referred to as ^Dox^R992C-ProGFP(+), and ^Dox^R992C-ProGFP(-), respectively. Mice developed in the absence of Dox are referred to as ^(-)Dox^R992C-ProGFP(+), and ^(-)Dox^R992C-ProGFP(-), respectively. Consequently, we define groups of mice based on the specific time point at which the mice started Dox treatmet and the age at which the mice were sacrificed.

For the 7-week-old mice, the following groups were analyzed: ^NB-Dox^R992C-ProGFP(+) (n = 3); ^NB-Dox^R992C-ProGFP(-) (n = 2); ^1w-Dox^R992C-ProGFP(+) (n = 2); ^1w-Dox^R992C-ProGFP(-) (n = 2); ^2w-Dox^R992C-ProGFP(+) (n = 6); ^2w-Dox^R992C-ProGFP(-) (n = 2); ^(-)Dox^R992C-ProGFP(+) (n = 7); ^(-)Dox^R992C-ProGFP(-) (n = 8).

For the 10-week-old mice, the following groups were analyzed: ^NB-Dox^R992C-ProGFP(+) (n = 3); ^NB-Dox^R992C-ProGFP(-) (n = 2); ^1w-Dox^R992C-ProGFP(+) (n = 4); ^1w-Dox^R992C-ProGFP(-) (n = 3); ^2w-Dox^R992C-ProGFP(+) (n = 2); ^2w-Dox^R992C-ProGFP(-) (n = 3); ^(-)Dox^R992C-ProGFP(+) (n = 8); ^(-)Dox^R992C-ProGFP(-) (n = 8).

Because chondrocyte proliferation assays, BiP content, and morphometry of bones of mice from the ^(-)Dox^R992C-ProGFP(+) and ^(-)Dox^R992C-ProGFP(-) groups were reported by us elsewhere, we did not carry out these assays here [25].

### Tissue collection and histology of growth plates

Mice were euthanized by CO_2_ overdose. Before processing the mice for whole-skeleton staining with alizarin red and alcian blue, their right hind limbs were collected for histology. Then, the hind paws were separated from the collected limbs. Next, the samples were fixed in a 4% solution of paraformaldehyde and then decalcified in a solution of 14% EDTA, pH = 7.1 for two weeks. To confirm that adding Dox inhibited the production of the R992C-ProGFP construct, decalcification was carried out in the dark in tubes wrapped in aluminum foil. This method prevented any GFP quenching that could occur if the R992C-ProGFP construct was produced [25]. The decalcified paws were embedded in the optimal cutting temperature compound (OCT; Sakura Finetek USA, Inc., Torrance, CA) for direct examination of GFP in analyzed tissues. The tibia-femur comples was embedded in paraffin [25].

The paraffin-embedded samples were stained with hematoxylin and eosin (H&E) to visualize the general morphology and the cellularity of the analyzed specimens. The samples were also stained with Sirius red (Polysciences Inc., Warrington, PA) to observe birefringent collagen fibrillar deposits with the use of a polarizing microscope (Eclipse LV100POL, Nikon Inc., Melville, NY). The OTC-embedded samples were cut into 20-μm slices and then stained with 4',6-diamidino-2-phenylindole (DAPI) to visualize the nuclei. Subsequently, these samples were observed with the use of a fluorescent microscope (Eclipse E600, Nikon Inc.) to confirm the absence of the R992C-ProGFP in the Dox-treated mice [25].

### Immunostaining of the growth plates

Tissue slices, 3-μm to 5-μm thick, of paraffin-embedded knee joints were processed for immunohistology, as described [25]. The following antigens were analyzed: (i) collagen X, a structural element of the hypertrophic zone; (ii) collagen VI, a structural protein from pericellular zone; (iii) binding immunoglobulin protein (BiP), a chaperon protein whose increased amount serves as an indicator of ER stress; and (iv) proliferating cell nuclear antigen (PCNA), employed as a marker of the division of chondrocytes.

To detect collagen X, we treated the samples with primary anti-collagen X antibody (Bioss Inc., Woburn, MA) followed by secondary biotinylated anti-rabbit IgG antibody (Vector Laboratories, Inc., Burlingame, CA). Collagen X-positive signals were then visualized with the use of horseradish peroxidase (HRP) and the NovaRED^™^ HRP substrate (Vector Laboratories, Inc.). Finally, the samples were counterstained by methyl green (Vector Laboratories, Inc.). Collagen VI was detected with the use of the anti-collagen VI antibody (Abcam, Cambridge, MA) and secondary anti-rabbit IgG antibody conjugated to Alexa Fluor 594 (Molecular Probes, Thermo Fisher Scientific, Inc.). For detection of BiP primary, anti-BiP antibody (Thermo Fisher Scientific, Inc.) and secondary anti-rabbit IgG antibody conjugated to Alexa Fluor 594 were employed. For immunostaining of PCNA, the tissues were first exposed to the anti-PCNA antibody (Thermo Fisher Scientific Inc.) and then to the biotinylated anti-mouse IgG secondary antibody (M.O.M. Kit, Vector laboratories, Inc.) and the streptavidin-conjugated Alexa Fluor 594. For the PCNA staining, specimens from the newborn mice were used as positive control. In all assays, we included negative controls in which primary antibodies against specific antigens were not used. A microscope (Eclipse E600, Nikon, Inc.), equipped with color and monochrome digital cameras (DS-Fi1 and DS-Qi1Mc, Nikon, Inc.), was employed to observe immunostained specimens.

### Image analyses

The main focus of microscopic assays was on the tibial growth plates. Microscopic images were processed with the use of the NIS Elements software (Nikon, Inc.). For specimens stained with fluorophores, a sequence of images of consecutive focal planes along the Z-axes of the analyzed areas was collected. Subsequently, these sequences were processed with the Extended Depth Focus module of the NIS Elements software (version Ar 4.5, Nikon, Inc.) to fully utilize in-focus information of the analyzed region of interest (ROI). A minimum of five slides from each analyzed specimen were prepared and analyzed.

### Hypertrophic zones

The height of the hypertrophic zones seen in H&E-stained specimens, also identified by the presence of collagen X in corresponding tissue sections, was measured using the NIS Elements software. These measurements were done within multiple ROIs covering the entire width of the tibial growth plates. To perform a measurement, a cursor was placed on the opposing borders of the hypertrophic zone to create two points defining a line. The points were placed so that the line was perpendicular to the opposing borders. Multiple lines were generated to cover the entire width of the analyzed growth plates. Subsequently, the lengths of the lines were recorded by the NIS Elements software.

### Acellular areas

The acellular regions surrounding groups of chondrocytes present in the tibial growth plates were readily visible in Sirius red-stained specimens observed with a polarizing microscope. Employing specific threshold values for the high and low intensities of the red, green, and blue colors in the RGB images enabled specific selection of the acellular areas. Finally, the acellular area was measured and then expressed as percent of the total area of the analyzed region.

### Proliferation of chondrocytes and BiP assays

The percentage of the PCNA-positive chondrocytes in the growth plates of the 7-week-old and 10-week-old mice was determined by quantitative fluorescence microscopy, as described by us [25]. BiP expression was analyzed in mature bones in 10-week-old mice. For each growth plate, a minimum of five sections were prepared and analyzed.

### Morphometric assays

The skeletal indices of the femora, tibiae, and skulls dissected from mice whose skeletons were stained with alizarin red and alcian blue were measured with a digital caliper to 0.01 mm (Absolute Digimatic Caliper, Mitutoyo Corporation, Kawasaki-shi, Japan), as described [25]. We analyzed only 10-week-old mice; at this age, skeletal growth and maturation are still ongoing, but the rates of these processes are low in comparison to the earlier stages [26–29]. The following parameters were recorded: (i) for the femora: the femoral length, the condylar, and the midshaft widths; (ii) for the tibiae: the tibia length and the condylar, the midshaft, and the malleolar widths; and (iii) for the skulls: the length, measured from the tip of the nasal bone to the back of the occipital bone, the width, measured at the widest point of the parietal bone, and the inner canthal distances. Subsequently, the length:mean-of-widths ratios were calculated to compare the shapes of the analyzed skeletal elements, as described [25]. Specifically, in comparing mutant mice with the relevant control group, a relatively small ratio was indicative of a disproportionately shorter bone.

### Data analysis

For histology-based assays of growth plates, we collected data from individual tissue sections derived from the R992C-ProGFP(+) and the R992C-ProGFP(-) mice. Specifically, we measured the average height of the hypertrophic zones, percent of area of growth plates occupied by the acellular regions, and the relative number of PCNA-positive cells. For each of the assays, we selected selected parameters within the representative areas of growth plates of analyzed samples.

We used linear regression to analyze the individual measurements for the height of hypertrophic zones and the percent of acellular areas. To account for the potential correlation of those measurements, since multiple measurements were contributed by each animal, we used the Generalized Estimating Equations (GEE) approach with the robust variance, *i*.*e*., p-values and confidence intervals were based on the GEE robust variance rather than the usual model-based variance that assumes independence.

Due to a relatively small number of samples available for the morphometry of bones, we report individual data points and the means with 95% confidence intervals (CI) for each analyzed group. Data analyses were done using the SAS software (version 9.4, SAS Institute Inc., Cary, NC).

## Results

### Transgenic mice and Dox treatment

In all assays, the R992C-ProGFP(+) mice and their R992C-ProGFP(-) control littermates were readily identified by PCR for the ProGFP, tTA, and Col2a1-cre constructs [25]. Consistent with our earlier studies, complete inhibition of the expression of the ProGFP constructs in all Dox-treated ^Dox^R992C-ProGFP(+) mice was confirmed by the absence of GFP-positive signals in chondrocytes (not shown) [25]. Although we did not directly measure the time from starting the Dox treatment to blocking the expression of the ProGFP in the ^Dox^R992C-ProGFP(+) mice, our earlier studies on chondrocytes isolated from the R992C-ProGFP(+) mice indicated that the expression stops after 48 h [25].

### Morphology of the growth plates

**Fig 1:**
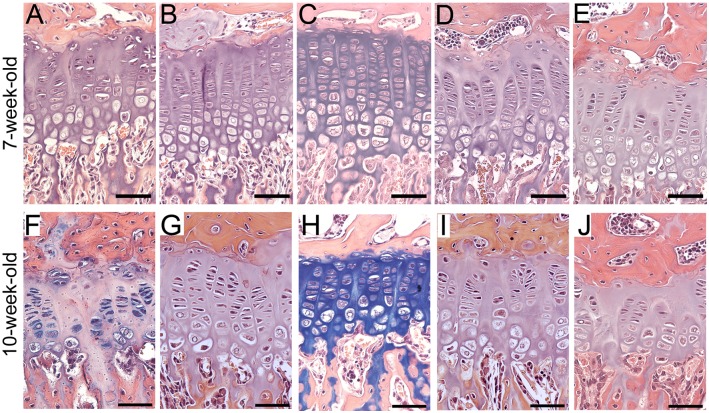
The general morphology of tibial growth plates of mice from the 7-week-old (A, B, C, D, and E) and 10-week-old (F, G, H, I, and J) groups. A&F, ^(-)Dox^R992C-ProGFP(+) mice in which expression of the R992C mutant is constant. B&G, ^(-)Dox^R992C-ProGFP(-) mice in which the mutant is not expressed. C&H, ^NB-Dox^R992C-ProGFP(+) mice. D&I, ^1w-Dox^R992C-ProGFP(+) mice. E&J, ^2w-Dox^R992C-ProGFP(+) mice. Bars = 50 μm.

Consistent with our earlier reports, chondrocytes in the growth plates of the R992C-ProGFP(+) mice from the 7-week-old and 10-week-old groups exhibited markedly altered arrangement and lacked well-defined columnar organization (Fig 1A & 1F). The increase of the area of the acellular regions was also clearly apparent. In contrast, in the corresponding R992C-ProGFP(-) mice, the chondrocytes were arranged in well-organized columns (Fig 1B & 1G). The area of the acellular regions seen in the growth plates was markedly smaller compared to the R992C-ProGFP(+) littermates.

In the 7-week-old and 10-week-old ^NB-Dox^R992C-ProGFP(+) mice, the morphology of the growth plates was similar to the corresponding R992C-ProGFP(-) control (Fig 1C & 1H). In contrast, chondrocytes in the growth plates of the ^1w-Dox^R992C-ProGFP(+) and ^2w-Dox^R992C-ProGFP(+) groups were noticeably disorganized (Fig 1D, 1E, 1I & 1J).

### Hypertrophic zones

**Fig 2:**
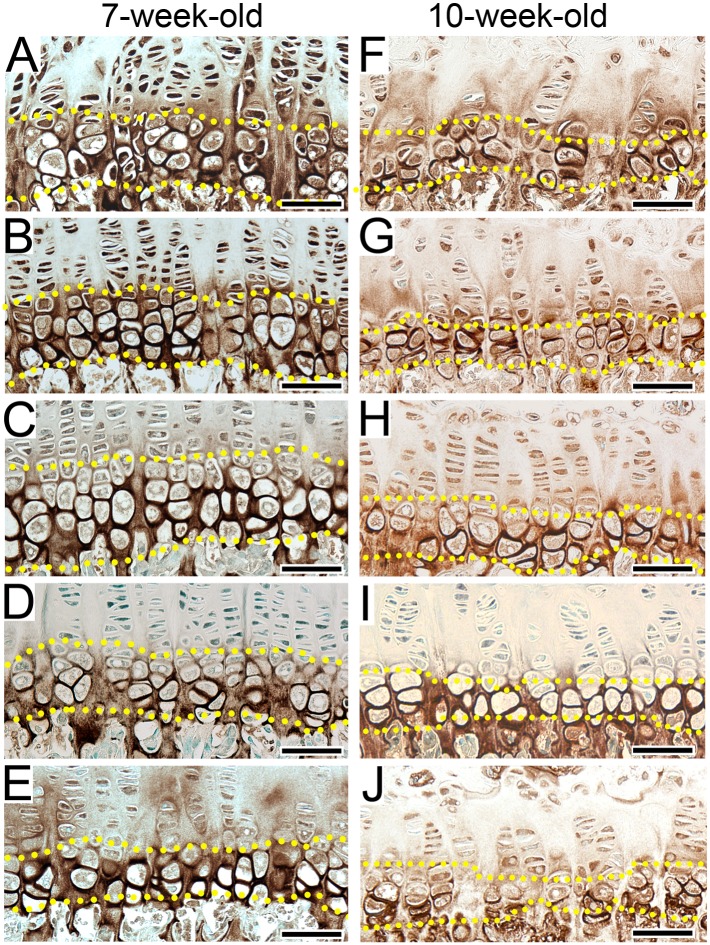
Immunostaining to detect collagen X in 7-week-old and 10-week-old mice. A&F, ^(-)Dox^R992C-ProGFP(+) mice in which expression of the R992C mutant is constant. B&G, ^(-)^DoxR992C-ProGFP(-) mice in which the mutant is not expressed. C&H, ^NB-Dox^R992C-ProGFP(+) mice. D&I, ^1w-Dox^R992C-ProGFP(+) mice. E&J, ^2w-Dox^R992C-ProGFP(+) mice. Dotted lines delineate the hypertrophic zones. Bars = 50 μm.

We observed that chondrocytes in the hypertrophic zones of the R992C-ProGFP(+) 7-week-old (Fig 2A) and 10-week-old (Fig 2F) mice were also disorganized compared to the R992C-ProGFP(-) littermates (Fig 2B & 2G). While the columnar organization improved in the ^NB-Dox^R992C-ProGFP(+) mice (Fig 2C & 2H), in the ^1w-Dox^R992C-ProGFP(+) and ^2w-Dox^R992C-ProGFP(+) groups chondrocytes appeared less organized (Fig 2D, 2E, 2I and 2J).

**Fig 3:**
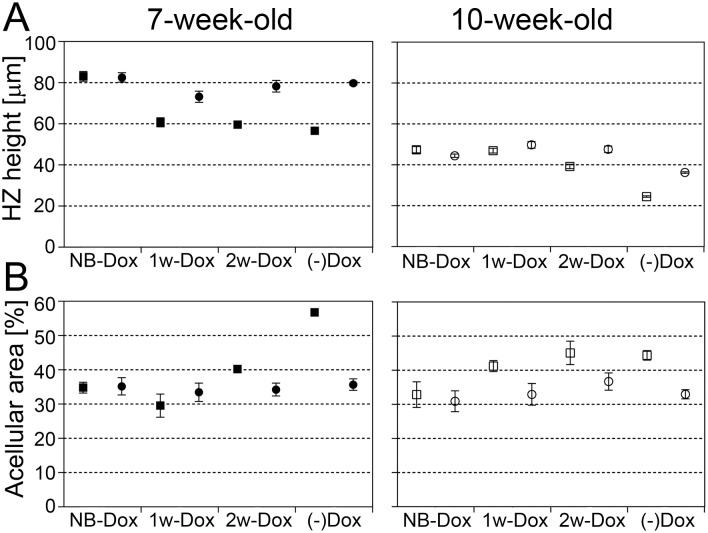
A graphic representation of measurements of the height of the hypertrophic zones and (A) the acellular areas (B). Data from the NB-Dox, the 1w-Dox, and the 2w-Dox mice from the 7-week-old and the 10-week-old age groups are presented. Data from the ^(-)Dox^R992C-ProGFP(+) and ^(-)Dox^R992C-ProGFP(-) mice are also shown (-Dox). The means with 95% CI are indicated. Symbols: ■ R992C-ProGFP(+) 7-week-old; ● R992C-ProGFP(-) 7-week-old; □ R992C-ProGFP(+) 10-week-old; ○ R992C-ProGFP(-) 10-week-old.

Measurements of the height of growth plates indicate a clear monotonic trend for the mean difference between the R992C-ProGFP(+) and the R992C-ProGFP(-) groups. Specifically, the R992C-ProGFP(+) values are much lower than the R992C-ProGFP(-) values when Dox is not given, and the difference diminishes as Dox is given earlier and earlier (Fig 3A and Table 1). The differences between the R992C-ProGFP(+) and the R992C-ProGFP(-) groups belonging to specific Dox-treatment categories, *i*.*e*. NB-Dox, 1w-Dox, 2w-Dox, or (-)Dox) are significantly different from each other (p = 0.001) and the trend test is also significant (p = 0.001) (Table 1).

**Table 1:** A summary of measurements of the heights of hypertrophic zones and the acellular areas of growth plates.

^a^^,^ ^b^**Measurements of the height of hypertrophic zones [μm] @ 7 weeks**				
Group	R992C-ProGFP(-)	R992C-ProGFP(+)	R992C-ProGFP(+) vs R992C-ProGFP(-)	p
	Mean	Mean	Mean difference	
**NB-Dox**	82.6	83.1	**0.5**	0.705
**1w-Dox**	73.2	60.8	**-12.4**	0.001
**2w-Dox**	78.3	59.6	**-18.7**	0.001
**(-)Dox**	79.8	56.7	**-23.1**	0.001
p (R992C-ProGFP(+) vs. R992C-ProGFP(-) difference vary?)				**0.001**
p (trend for R992C-ProGFP(+) vs. R992C-ProGFP(-) difference?)				**0.001**
^c^^,^ ^d^**Measurements of the height of hypertrophic zones [μm] @ 10 weeks**				
Group	R992C-ProGFP(-)	R992C-ProGFP(+)	R992C-ProGFP(+) vs. R992C-ProGFP(-)	p
	Mean	Mean	Mean difference	
**NB-Dox**	44.5	47.4	**2.9**	0.004
**1w-Dox**	49.8	46.9	**-2.9**	0.195
**2w-Dox**	47.7	39.2	**-8.5**	0.001
**(-)Dox**	36.3	24.5	**-11.8**	0.001
p (R992C-ProGFP(+) vs. R992C-ProGFP(-) difference vary?)				**0.001**
p (trend for R992C-ProGFP(+) vs. R992C-ProGFP(-) difference?)				**0.001**
^a^^,^ ^e^**Measurements of the acellular areas [%] @ 7 weeks**				
Group	R992C-ProGFP(-)	R992C-ProGFP(+)	R992C-ProGFP(+) vs. R992C-ProGFP(-)	p
	Mean	Mean	Mean difference	
**NB-Dox**	35.2	34.8	**-0.4**	0.538
**1w-Dox**	33.5	29.6	**-3.9**	0.004
**2w-Dox**	34.2	40.2	**6.0**	0.001
**(-)Dox**	35.7	56.8	**21.1**	0.001
p (R992C-ProGFP(+) vs. R992C-ProGFP(-) difference vary?)				**0.001**
p (trend for R992C-ProGFP(+) vs. R992C-ProGFP(-) difference?)				**0.001**
^c^^,^ ^f^**Measurements of the acellular areas [%] @ 10 weeks**				
Group	R992C-ProGFP(-)	R992C-ProGFP(+)	R992C-ProGFP(+) vs. R992C-ProGFP(-)	p
	Mean	Mean	Mean difference	
**NB-Dox**	30.9	33.0	**2.1**	0.120
**1w-Dox**	33.0	41.2	**8.2**	0.001
**2w-Dox**	36.7	45.1	**8.4**	0.001
**(-)Dox**	33.0	44.4	**11.4**	0.001
p (R992C-ProGFP(+) vs. R992C-ProGFP(-) difference vary?)				**0.001**
p (trend for R992C-ProGFP(+) vs. R992C-ProGFP(-) difference?)				**0.001**

^a^32 mice were analyzed: 14 R992C-ProGFP(-), 18 R992C-ProGFP(+).

^b^773 measurements were done (average = 24 measurements/mouse).

^c^33 mice were analyzed: 16 R992C-ProGFP(-), 17 R992C-ProGFP(+).

^d^802 measurements were done (average = 24 measurements/mouse).

^e^304 measurements were done (average = 10 measurements/mouse).

^f^314 measurements were done (average = 10 measurements/mouse).

### Area of the acellular space

**Fig 4:**
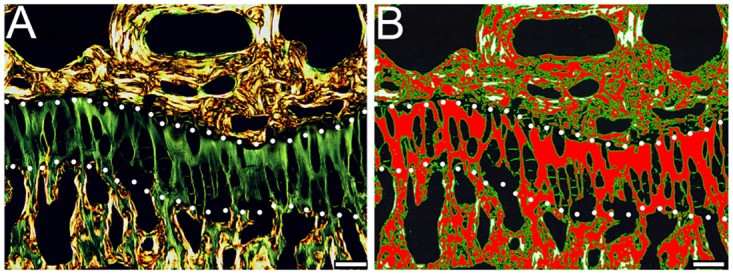
A representative image of a Sirius red-stained tibial growth plate observed in polarized light. A, The acellular area of the growth plate is readily identified by green-color birefringence. B, The acellular area is segregated from the rest of the growth plate by a specific intensity threshold indicated by red color. The acellular area is calculated as a percent of the total area of a growth plate delineated in B.

Measurements of the acellular areas defined by Sirius red staining of growth plates (Fig 4) corroborated the morphological changes observed in analyzed samples (Fig 1). These measurements indicate a monotonic trend for the mean difference between R992C-ProGFP(+) and R992C-ProGFP(-) groups, with R992C-ProGFP(+) values being higher than R992C-ProGFP(-) values when Dox is not given. The difference decreases as Dox is given earlier and earlier. The trend is not as clean, however, particularly for the 7-weeks data (Fig 3B and Table 1). Similar to the results on the height of the hypertrophic zones, the differences between the R992C-ProGFP(+) and the R992C-ProGFP(-) groups belonging to specific Dox-treatment categories, *i*.*e*. NB-Dox, 1w-Dox, 2w-Dox, or (-)Dox) are significantly different from each other (p = 0.001) and the trend test is also significant (p = 0.001) (Table 1).

### Pericellular matrix

**Fig 5:**
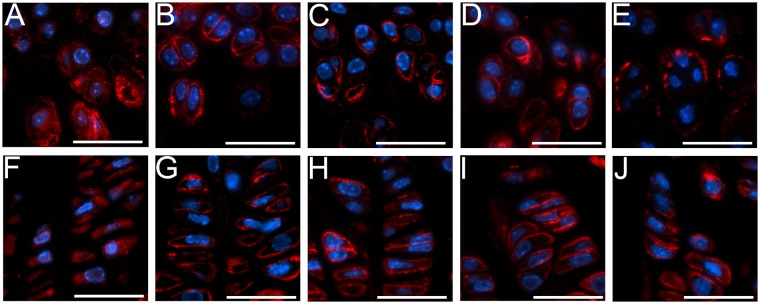
Immunostaining of collagen VI in 10-week-old mice. A, B, C, D, and E, collagen VI distribution in articular cartilage. F, G, H, I, and J, collagen VI distribution in growth plates. A&F, ^(-)Dox^R992C-ProGFP(+) mice with constant expression of the R992C mutant. B&G, ^(-)Dox^R992C-ProGFP(-) mice in which the mutant is not expressed. C&H, ^NB-Dox^R992C-ProGFP(+) mice. D&I, ^1w-Dox^R992C-ProGFP(+) mice. E&J, ^2w-Dox^R992C-ProGFP(+) mice. Bars = 25 μm.

To analyze the impact of the R992C-ProGFP on the organization of pericellular matrix, we analyzed the distribution of collagen VI in articular cartilage and growth plates. For these assays we selected 10-week-old mice in which the bones had reached maturity. As indicated in Fig 5A, the distribution of collagen VI around chondrocytes present in articular cartilage of the ^(-)Dox^R992C-ProGFP(+) mice was diffused. Intracellular localization of collagen VI was also apparent. In contrast, in the corresponding regions seen in the ^(-)Dox^R992C-ProGFP(-) control mice, collagen VI was located within well-defined pericellular area (Fig 5B). In the ^NB-Dox^R992C-ProGFP(+) mice (Fig 5C), the pattern of distribution of collagen VI was similar to that of control, while in the ^1w-Dox^R992C-ProGFP(+) and ^2w-Dox^R992C-ProGFP(+) mice, collagen VI was distributed in diffused pattern (Fig 5D & 5E). Moreover, in the ^2w-Dox^R992C-ProGFP(+) mice, instead of forming a defined, uniform ring around chondrocytes, collagen VI showed a “spotted” distribution pattern (Fig 5E). Similar alterations of collagen VI distribution were observed around chondrocytes present in the growth plates (Fig 5F, 5G, 5H, 5I & 5J)

### Expression of PCNA and BiP

Quantification of PCNA-positive chondrocytes in the 7-week-old and 10-week-old groups demonstrated no differences between their relative numbers in the growth plates of the ^NB-Dox^R992C-ProGFP(+) and the ^NB-Dox^R992C-ProGFP(-) groups, the ^1w-Dox^R992C-ProGFP(+) and the ^1w-Dox^R992C-ProGFP(-) groups, or the ^2w-Dox^R992C-ProGFP(+) and the ^2w-Dox^R992C-ProGFP(-) groups (data not shown).

**Fig 6:**
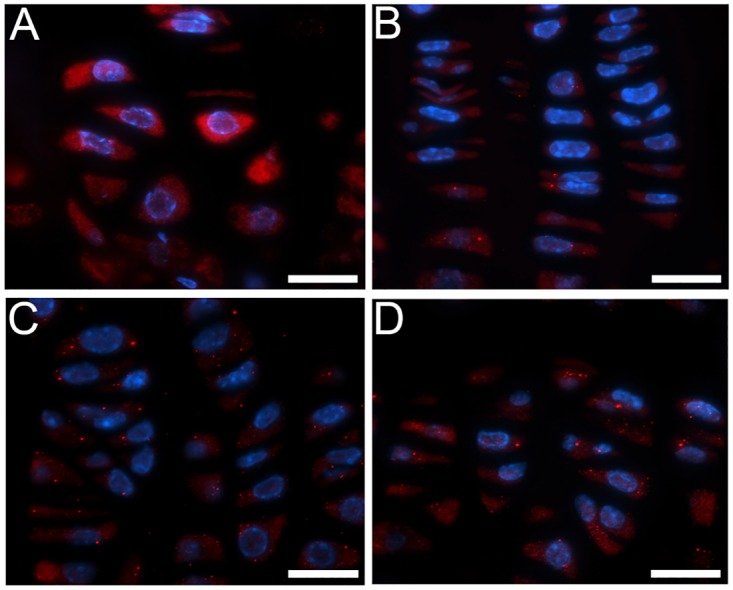
BiP-specific immunostaining of growth plates of 10-week-old mice. A, ^(-)Dox^R992C-ProGFP(+) mice; B, ^NB-Dox^R992C-ProGFP(+) mice; C, ^1w-Dox^R992C-ProGFP(+) mice; D, ^2w-Dox^R992C-ProGFP(+) mice. Bars = 20 μm.

The BiP-positive chondrocytes were readily apparent in the ^(-)Dox^R992C-ProGFP(+) control mice with the constant expression of the R992C collagen II (Fig 6A), but this chaperone was essentially absent in the ^NB-Dox^R992C-ProGFP(+), ^1w-Dox^R992C-ProGFP, and ^2w-Dox^R992C-ProGFP(+) groups (Fig 6B, 6C & 6D).

### Morphometry of bones

**Fig 7:**
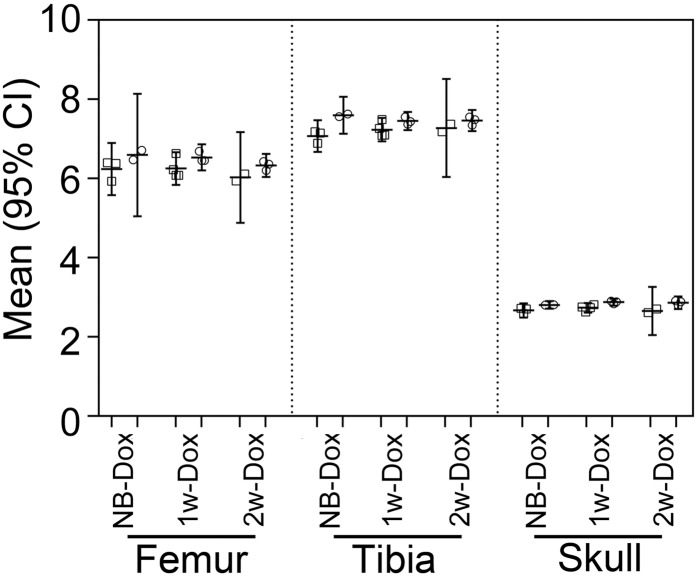
A graphic representation of differences between the length:width ratios of femora, tibiae, and skulls of the 10-week-old mice maintained in the presence of Dox added postnatally. The individual data points and the means with 95% CI are presented. Symbols: □ R992C-ProGFP(+);○ R992C-ProGFP(-).

We measured the length:mean-of-widths ratios as useful indices to compare the shapes of the analyzed skeletal elements; a relatively small ratio indicated a disproportionately shorter bone. Specifically, we measured these ratios for the femora, tibiae, and skulls of the 10-week-old ^NB-Dox^R992C-ProGFP(+),^1w-Dox^R992C-ProGFP(+), and ^2w-Dox^R992C-ProGFP(+) mice. Then we compared these indices to those measured for the respective R992C-Pro-GFP(-) controls. In all groups of the R992C-ProGFP(+) mice, these indices trended toward lower values compared to the R992C-ProGFP(-) controls (Fig 7).

## Discussion

In our earlier studies on the mild form of SED caused by the R992C collagen II, we demonstrated perturbations of the growth plates in mice harboring this mutant [25]. This research showed aberrant ECM formed in the presence of the mutant molecules. Specifically, we demonstrated poor organization of collagen II-rich assemblies formed within interterritorial matrix that separates the columns of chondrocytes. Moreover, we observed alteration of collagen X deposits present in the hypertrophic zones [25]. We also demonstrated that changes in the ECM alter the primarily cilia-mediated spatial organization of chondrocytes and that excessive intracellular accumulation of collagen II and ER stress reduced proliferation of chondrocytes. We clearly showed that these changes were trigerred by the presence of the R992C mutant collagen II. Specifically, we demonstrated that switching off the expression of the R992C collagen II during the entire period of prenatal and postnatal development blocks the formation of the SED phenotype [25].

Here, we tested the ability of the growth plates formed in the presence of the R992C collagen II to rearrange their aberrant organization toward normal architecture upon postnatal inhibition of the expression of the R992C collagen II. After switching off the expression in newborn, 1-week-old, and 2-week-old mice, we analyzed their growth plates after they reached 7 weeks or 10 weeks of age. To determine the effects of blocking the expression of the R992C collagen II, we analyzed the morphology of the tibial growth plates and studied selected cellular parameters that define cell proliferation and ER stress. We also analyzed the shapes of femora, tibiae, and skulls in 10-week-old mice that reached skeletal maturity.

Our studies clearly demonstrated that the morphology of the growth plates improved significantly only when the production of the R992C mutant collagen II was switched off in newborn mice. Blocking the expression of the R992C collagen II at later postnatal stages had less significant effects.

A perturbed arrangement of columns of chondrocytes was readily visible in the 7-week-old and the 10-week-old mice from the ^1w-Dox^R992C-ProGFP(+) and the ^2w-Dox^R992CProGFP(+) groups. This abnormal arrangement was highlighted by the increased size of the acellular areas among groups of chondrocytes.

In addition to the perturbed columnar arrangement of chondrocytes within the proliferative zones, we observed a significant decrease in the height of the hypertrophic zones in the ^1w-Dox^R992C-ProGFP(+) and the ^2w-Dox^R992C-ProGFP(+) mice from the 7-week-old and 10-week-old groups. Since the height of hypertrophic chondrocytes, their correct spatial organization, and proper matrix deposition are key elements of skeletal growth, the inability to fully restore these characteristics may contribute to the persistence of bone aberrations even after the elimination of the R992C collagen II [30].

In our earlier studies, we demonstrated that misfolding of the R992C collagen II molecules delays their secretion from chondrocytes, thus causing excessive intacellular accumulation of this mutant. We showed that pathological effects of this accumulation were associated with ER stress [25, 31, 32]. By showing that inhibiting the expression of the R992C mutant from the beginning of the embryonic development prevents the formation of the SED phenotype, we clearly associated ER stress with pathomechanisms of this disease [25]. Here, we demonstrated a marked reduction of BiP, which indicates elimination of ER stress in the ^NB-Dox^R992C-ProGFP(+), the ^1w-Dox^R992C-ProGFP(+), and the ^2w-Dox^R992C-ProGFP(+) groups. Even when ER stress was eliminated, however, the abnormal morphology of the growth plates persisted in skeletally mature mice from these groups. Thus, we contemplate that the main underlying factor that hampered growth plate remodeling after switching off the production of the R992C mutant was the stability of aberrant architecture of the ECM formed prior to making this switch.

Our studies indicate that the growth plates developed in mice in the presence of R992C collagen II have only a limited ability to remodel into the correct structure if the mutant expression is not blocked until after bone growth is in the the postnatal stages. Our studies showed that the extent of improvements of measured parameters was clearly a function of when the expression of R992C collagen II was switched off. The most significant improvements were observed in the ^NB-Dox^R992C-ProGFP(+) group. In this group, the inhibition of the expression of R992C collagen II was initiated before the start of a period of a rapid postnatal growth of the long bones. In a number of strains of laboratory mice, this period spans day 7 to 23 after birth [33]. Despite these morphological improvements, our results suggest that the shapes of bones in the ^NB-Dox^R992C-ProGFP(+) mice did not reach the parameters of the normal controls and were comparable to those observed in the ^1w-Dox^R992C-ProGFP(+) and ^2w-Dox^R992C-ProGFP(+) groups. In contrast, our earlier study demonstrated that switching off the expression of the R992C mutant through the entire prenatal and postnatal development leads to development of bones with morphomethic indices identical to those of wild type controls [25].

We contemplate that the inability to improve the morphometric parameters of femora, tibiae, and skulls was a result of irreversible changes to the architecture of early cartilaginous matrices that define the shape of bones. While the shapes of femora and tibiae are largely decided by the activity of their growth plates, the shape of a mouse skull is influenced by the development of the cranial base. Although the vault of a skull is formed by the intramembranous bones, the cranial base is formed by endochondral bones. This base is shaped by activities of synchondroses that, fundamentally, are similar to the epiphyseal growth plates. Because in mice the cranial base spans almost the entire rostro-caudal length, its proper development is critical for the growth of the skull as a whole [34].

The observation that most effective remodeling of growth plates formed in the presence of the R992C collagen II mutant was possible only if the expression of this mutant was switched off right after birth is consistent with a study recently published by Prein *et al*. [35]. In this study, the authors demonstrated proper fibrillar architecture and mechanical characteristics of the interterritorial and pericellular collagenous matrices are critical for a correct columnar arrangement of chondrocytes that form the growth plate. The authors showed that collagen fibrils that form key architectural elements of the interterritorial and pericellular matrices start to arrange from day E 13.5, and this process is essentially completed when mice are 2 weeks old. From this point, no major changes in fibrillar architecture take place. Furthemore, the authors demonstrated that the mechanical properties of the collagenous matrices of the interterritorial and the pericellular matrices also stabilize in 2-week-old mice.

Considering the complexity of collagenous matrix formed by co-assembly of collagen II and collagen XI as well as by binding interactions of these heterotypic fibrils with collagen IX and proteoglycans, we propose that the presence of the R992C mutant alters the matrix’s architecture. In addition to our earlier observations showing aberrations of collagenous matrix in growth plates of the R992C-ProGFP(+) mice, here we demonstrated changes in the pericellular organization of collagen VI. In normal cartilage, collagen VI is found in pericellular matrix that that envelopes chondrocytes. This protein is a part of the pericellular microenvironment, the chondron, that mediates interaction between the chondrocyte and its ECM [36]. Researchers demonstrated that, in its outer margin, collagen VI interacts with collasgen II fibrils, thereby providing a contact between the pericellular and territorial matrices [37]. We propose that diffused pattern of distribution of collagen VI in the R992C-ProGFP(+) mice may result from poor architecture of collagen II-rich matrix altered by the presence of the R992C collagen II mutant.

In earlier studies, we demonstrated aberrations of collagen fibrils present in the interterritorial space and showed increased intracellular accumulation of the R992C mutant [23, 25, 31]. Our studies demonstrated that this accumulation was a result of delayed secretion of this mutant due to its lower thermostability [31]. Here, we also observed atypical accumulation of collagen VI in chondrocytes seen in the R992C-ProGFP(+) mice. This observation may suggest that collagen VI is trapped inside cells due to its atypical binding to partially unfolded R992C-ProGFP mutant [23, 31]. Earlier, we observed a similar intracellular trapping of fibronectin by the misfolded R789C collagen II mutant associated with the lethal form of SED [5, 38]. Intracellular entrapment of normal molecules destined for secretion due to the presence of mutant proteins is not unique to collagen II. For instance, Merritt *et al*. have reported intracellular aggregation of normal collagen II, collagen IX, and matrilin 3 due to the presence of mutant cartilage oligomeric matrix protein in chondrocytes derived from PSACH patients [39].

The observations on aberrations of collagen VI distribution further indicate far-reaching consequences of the R992C mutation in collagen II and point toward the extensive damage that this mutation causes in the structure of the ECM of cartilage.

Our findings that switching off the expression of the R992C mutant in 1-week-old and in 2-week-old R992C-ProGFP(+) mice does not restore proper organization of collagen VI further suggests that, at these postnatal stages, the ability of the ECM formed in the presence of the R992C collagen II mutant is limited.

In this context, we contemplate that therapeutic approaches for targeting heritable skeletal dysplasias have a better chance of success when they aim to block the onset of pathological changes rather than to repair their consequences. This postulation is in agreement with work demonstrating the curative effects of statins applied at early postnatal stages in a mouse model of ACH caused by mutations in the fibroblast growth factor receptor 3 gene (*Fgfr3*) [7]. Moreover, our postulation is in agreement with studies on a mouse model of Alport syndrome caused by the absence of the α3(IV) chain. In this model Lin *et al*. demonstrated that switching on the expression of this missing chain prenatally or within three weeks after birth positively affected the architecture and function of glomerular basement membrane. Within this time window, when mouse glomeruli are still formed, a functional collagen IV network was formed despite the existence of abnormal structures assembled earlier in the absence of the α3(IV) chain [40].

Considering that the complete prevention of the SED phenotype was possible in our model when R992C collagen II expression was blocked at the beginning of embryonic development, and, to a certain extent in newborn mice, our study validates the pursuit of therapeutic approaches.

Our study has a few limitations. First, the number of samples available for morphometry that showed a trend toward shortening of bones was limited because only 12.5% of littermates expressed the three transgenes needed for the production of the R992C mutant. Thus, although we observed differences between the R992C-ProGFP(+) mice and their R992C-ProGFP(-) littermates, the statistical significance of these changes could not be determined. Second, our experimental system did not allow us to directly measure the amount of mutant collagen II that could still be present in mature cartilage of the NB-Dox, 1w-Dox, and 2w-Dox mice. We know, however, based on studies using cell-based models of SED and on certain cases of OI, that the diseased phenotype may occur even when the content of the mutant chains is below 10% of the total collagen content [32, 41]. Third, since strict correlations between mice and human developmental time lines do not exist, based on this study we cannot clearly determine the most optimal time frame for effective therapeutic interventions to limit the effects of collagen II mutations harbored by SED patients. Still, this study suggests that future therapies of skeletal dysplasias may require defining a specific time window when interventions should be applied to be successful.

## References

[1] Lefebvre V, Bhattaram P. Vertebrate skeletogenesis. Current topics in developmental biology. 2010;90:291–317. Epub 2010/08/10. 10.1016/S0070-2153(10)90008-2 20691853 PMC3077680

[2] Mackie EJ, Tatarczuch L, Mirams M. The skeleton: a multi-functional complex organ: the growth plate chondrocyte and endochondral ossification. The Journal of endocrinology. 2011;211(2):109–21. Epub 2011/06/07. 10.1530/JOE-11-0048 21642379

[3] Arnold WV, Fertala A. Skeletal diseases caused by mutations that affect collagen structure and function. Int J Biochem Cell Biol. 2013;45(8):1556–67. Epub 2013/05/28. 10.1016/j.biocel.2013.05.017 23707199

[4] Fertala A, Sieron AL, Adachi E, Jimenez SA. Collagen II containing a Cys substitution for Arg-alpha1-519: abnormal interactions of the mutated molecules with collagen IX. Biochemistry. 2001;40(48):14422–8. 11724554 10.1021/bi0109109

[5] Ito H, Rucker E, Steplewski A, McAdams E, Brittingham RJ, Alabyeva T, et al Guilty by association: some collagen II mutants alter the formation of ECM as a result of atypical interaction with fibronectin. J Mol Biol. 2005;352(2):382–95. 10.1016/j.jmb.2005.07.019 16083907

[6] Kanazawa H, Tanaka H, Inoue M, Yamanaka Y, Namba N, Seino Y. Efficacy of growth hormone therapy for patients with skeletal dysplasia. Journal of bone and mineral metabolism. 2003;21(5):307–10. Epub 2003/08/21. 10.1007/s00774-003-0425-7 12928832

[7] Yamashita A, Morioka M, Kishi H, Kimura T, Yahara Y, Okada M, et al Statin treatment rescues FGFR3 skeletal dysplasia phenotypes. Nature. 2014;513(7519):507–11. Epub 2014/09/19. 10.1038/nature13775 25231866

[8] Horwitz EM, Prockop DJ, Gordon PL, Koo WW, Fitzpatrick LA, Neel MD, et al Clinical responses to bone marrow transplantation in children with severe osteogenesis imperfecta. Blood. 2001;97(5):1227–31. 11222364 10.1182/blood.v97.5.1227

[9] Horwitz EM, Prockop DJ, Fitzpatrick LA, Koo WW, Gordon PL, Neel M, et al Transplantability and therapeutic effects of bone marrow-derived mesenchymal cells in children with osteogenesis imperfecta. Nat Med. 1999;5(3):309–13. 10.1038/6529 10086387

[10] Horwitz EM, Gordon PL, Koo WK, Marx JC, Neel MD, McNall RY, et al Isolated allogeneic bone marrow-derived mesenchymal cells engraft and stimulate growth in children with osteogenesis imperfecta: Implications for cell therapy of bone. Proc Natl Acad Sci U S A. 2002;99(13):8932–7. 10.1073/pnas.132252399 12084934 PMC124401

[11] Le Blanc K, Gotherstrom C, Ringden O, Hassan M, McMahon R, Horwitz E, et al Fetal mesenchymal stem-cell engraftment in bone after in utero transplantation in a patient with severe osteogenesis imperfecta. Transplantation. 2005;79(11):1607–14. Epub 2005/06/09. 15940052 10.1097/01.tp.0000159029.48678.93

[12] Gotherstrom C, Westgren M, Shaw SW, Astrom E, Biswas A, Byers PH, et al Pre- and postnatal transplantation of fetal mesenchymal stem cells in osteogenesis imperfecta: a two-center experience. Stem cells translational medicine. 2014;3(2):255–64. Epub 2013/12/18. 10.5966/sctm.2013-0090 24342908 PMC3925052

[13] Chan JK, Gotherstrom C. Prenatal transplantation of mesenchymal stem cells to treat osteogenesis imperfecta. Frontiers in pharmacology. 2014;5:223 Epub 2014/10/28. 10.3389/fphar.2014.00223 25346689 PMC4191163

[14] Millington-Ward S, McMahon HP, Farrar GJ. Emerging therapeutic approaches for osteogenesis imperfecta. Trends Mol Med. 2005;11(6):299–305. Epub 2005/06/14. 10.1016/j.molmed.2005.04.006 15949772

[15] Dawson PA, Marini JC. Hammerhead ribozymes selectively suppress mutant type I collagen mRNA in osteogenesis imperfecta fibroblasts. Nucleic Acids Res. 2000;28(20):4013–20. 11024182 10.1093/nar/28.20.4013 PMC110781

[16] Peace BE, Florer JB, Witte D, Smicun Y, Toudjarska I, Wu G, et al Endogenously expressed multimeric self-cleaving hammerhead ribozymes ablate mutant collagen in cellulo. Mol Ther. 2005;12(1):128–36. 10.1016/j.ymthe.2005.02.015 15963928

[17] Wang Q, Marini JC. Antisense oligodeoxynucleotides selectively suppress expression of the mutant alpha 2(I) collagen allele in type IV osteogenesis imperfecta fibroblasts. A molecular approach to therapeutics of dominant negative disorders. J Clin Invest. 1996;97(2):448–54. 10.1172/JCI118434 8567966 PMC507036

[18] Forlino A, Cabral WA, Barnes AM, Marini JC. New perspectives on osteogenesis imperfecta. Nature reviews Endocrinology. 2011;7(9):540–57. Epub 2011/06/15. 10.1038/nrendo.2011.81 21670757 PMC3443407

[19] Rochet JC. Novel therapeutic strategies for the treatment of protein-misfolding diseases. Expert reviews in molecular medicine. 2007;9(17):1–34. Epub 2007/06/29. 10.1017/S1462399407000385 17597554

[20] Sloan LA, Fillmore MC, Churcher I. Small-molecule modulation of cellular chaperones to treat protein misfolding disorders. Curr Opin Drug Discov Devel. 2009;12(5):666–81. Epub 2009/09/09. 19736625

[21] Briggs MD, Bell PA, Wright MJ, Pirog KA. New therapeutic targets in rare genetic skeletal diseases. Expert opinion on orphan drugs. 2015;3(10):1137–54. Epub 2015/12/05. 10.1517/21678707.2015.1083853 26635999 PMC4643203

[22] Posey KL, Coustry F, Veerisetty AC, Liu P, Alcorn JL, Hecht JT. Chondrocyte-specific pathology during skeletal growth and therapeutics in a murine model of pseudoachondroplasia. J Bone Miner Res. 2014;29(5):1258–68. Epub 2013/11/07. 10.1002/jbmr.2139 24194321 PMC4075045

[23] Gawron K, Jensen DA, Steplewski A, Fertala A. Reducing the effects of intracellular accumulation of thermolabile collagen II mutants by increasing their thermostability in cell culture conditions. Biochem Biophys Res Commun. 2010;396(2):213–8. Epub 2010/04/17. 10.1016/j.bbrc.2010.04.056 20394730 PMC2878901

[24] Okada M, Ikegawa S, Morioka M, Yamashita A, Saito A, Sawai H, et al Modeling type II collagenopathy skeletal dysplasia by directed conversion and induced pluripotent stem cells. Hum Mol Genet. 2015;24(2):299–313. Epub 2014/09/05. 10.1093/hmg/ddu444 25187577

[25] Arita M, Fertala J, Hou C, Steplewski A, Fertala A. Mechanisms of Aberrant Organization of Growth Plates in Conditional Transgenic Mouse Model of Spondyloepiphyseal Dysplasia Associated with the R992C Substitution in Collagen II. The American journal of pathology. 2015;185(1):214–29. Epub 2014/12/03. 10.1016/j.ajpath.2014.09.003 25451152

[26] Somerville JM, Aspden RM, Armour KE, Armour KJ, Reid DM. Growth of C57BL/6 mice and the material and mechanical properties of cortical bone from the tibia. Calcified tissue international. 2004;74(5):469–75. Epub 2004/02/13. 10.1007/s00223-003-0101-x 14961209

[27] Brodt MD, Ellis CB, Silva MJ. Growing C57Bl/6 mice increase whole bone mechanical properties by increasing geometric and material properties. J Bone Miner Res. 1999;14(12):2159–66. Epub 2000/01/05. 10.1359/jbmr.1999.14.12.2159 10620076

[28] Chambers MG, Kuffner T, Cowan SK, Cheah KS, Mason RM. Expression of collagen and aggrecan genes in normal and osteoarthritic murine knee joints. Osteoarthritis Cartilage. 2002;10(1):51–61. Epub 2002/02/14. 10.1053/joca.2001.0481 11795983

[29] Kveiborg M, Albrechtsen R, Rudkjaer L, Wen G, Damgaard-Pedersen K, Wewer UM. ADAM12-S stimulates bone growth in transgenic mice by modulating chondrocyte proliferation and maturation. J Bone Miner Res. 2006;21(8):1288–96. Epub 2006/07/28. 10.1359/jbmr.060502 16869727

[30] Farquharson C, Jefferies D. Chondrocytes and longitudinal bone growth: the development of tibial dyschondroplasia. Poultry science. 2000;79(7):994–1004. Epub 2000/07/20. 10901201 10.1093/ps/79.7.994

[31] Chung HJ, Jensen DA, Gawron K, Steplewski A, Fertala A. R992C (p.R1192C) Substitution in collagen II alters the structure of mutant molecules and induces the unfolded protein response. J Mol Biol. 2009;390(2):306–18. Epub 2009/05/13. 10.1016/j.jmb.2009.05.004 19433093 PMC2749300

[32] Jensen DA, Steplewski A, Gawron K, Fertala A. Persistence of intracellular and extracellular changes after incompletely suppressing expression of the R789C (p.R989C) and R992C (p.R1192C) collagen II mutants. Hum Mutat. 2011;32(7):794–805. Epub 2011/04/08. 10.1002/humu.21506 21472893 PMC4246508

[33] Richman C, Kutilek S, Miyakoshi N, Srivastava AK, Beamer WG, Donahue LR, et al Postnatal and pubertal skeletal changes contribute predominantly to the differences in peak bone density between C3H/HeJ and C57BL/6J mice. J Bone Miner Res. 2001;16(2):386–97. Epub 2001/02/24. 10.1359/jbmr.2001.16.2.386 11204439

[34] McBratney-Owen B, Iseki S, Bamforth SD, Olsen BR, Morriss-Kay GM. Development and tissue origins of the mammalian cranial base. Dev Biol. 2008;322(1):121–32. Epub 2008/08/06. 10.1016/j.ydbio.2008.07.016 18680740 PMC2847450

[35] Prein C, Warmbold N, Farkas Z, Schieker M, Aszodi A, Clausen-Schaumann H. Structural and mechanical properties of the proliferative zone of the developing murine growth plate cartilage assessed by atomic force microscopy. Matrix Biol. 2016;50:1–15. Epub 2015/10/11. 10.1016/j.matbio.2015.10.001 26454027

[36] Poole CA, Flint MH, Beaumont BW. Chondrons in cartilage: ultrastructural analysis of the pericellular microenvironment in adult human articular cartilages. J Orthop Res. 1987;5(4):509–22. Epub 1987/01/01. 10.1002/jor.1100050406 3681525

[37] Poole CA, Ayad S, Gilbert RT. Chondrons from articular cartilage. V. Immunohistochemical evaluation of type VI collagen organisation in isolated chondrons by light, confocal and electron microscopy. J Cell Sci. 1992;103 (Pt 4):1101–10. Epub 1992/12/01. 1487492 10.1242/jcs.103.4.1101

[38] Chan D, Taylor TK, Cole WG. Characterization of an arginine 789 to cysteine substitution in alpha 1 (II) collagen chains of a patient with spondyloepiphyseal dysplasia. J Biol Chem. 1993;268(20):15238–45. 8325895

[39] Merritt TM, Bick R, Poindexter BJ, Alcorn JL, Hecht JT. Unique matrix structure in the rough endoplasmic reticulum cisternae of pseudoachondroplasia chondrocytes. The American journal of pathology. 2007;170(1):293–300. 10.2353/ajpath.2007.060530 17200202 PMC1762700

[40] Lin X, Suh JH, Go G, Miner JH. Feasibility of repairing glomerular basement membrane defects in Alport syndrome. J Am Soc Nephrol. 2014;25(4):687–92. Epub 2013/11/23. 10.1681/ASN.2013070798 24262794 PMC3968506

[41] Cabral WA, Fertala A, Green LK, Korkko J, Forlino A, Marini JC. Procollagen with skipping of alpha 1(I) exon 41 has lower binding affinity for alpha 1(I) C-telopeptide, impaired in vitro fibrillogenesis, and altered fibril morphology. J Biol Chem. 2002;277(6):4215–22. 10.1074/jbc.M109048200 11706004

